# Dysregulated T Cell Activation and Aberrant Cytokine Expression Profile in Systemic Lupus Erythematosus

**DOI:** 10.1155/2019/8450947

**Published:** 2019-03-17

**Authors:** Haiyan Zhou, Bojiang Li, Jing Li, Tongqian Wu, Xiaoqian Jin, Rui Yuan, Ping Shi, Yan Zhou, Long Li, Fang Yu

**Affiliations:** ^1^Clinical Research Center, The Affiliated Hospital of Guizhou Medical University, Guiyang 550004, China; ^2^Guizhou Medical University, Guiyang 550004, China; ^3^Department of Immunology and Rheumatology, The Affiliated Hospital of Guizhou Medical University, Guiyang 550004, China; ^4^Clinical Laboratory Center, The Affiliated Hospital of Guizhou Medical University, Guiyang 550004, China; ^5^Department of Immunology and Rheumatology, The Third Affiliated Hospital of Guizhou Medical University, Guiyang 550004, China

## Abstract

Accumulating evidence indicates a critical role for T cells and relevant cytokines in the pathogenesis of systemic lupus erythematosus (SLE). However, the specific contribution of T cells together with the related circulating cytokines in disease pathogenesis and organ involvement is still not clear. In the current study, we investigated relevant molecule expressions and cytokine levels in blood samples from 49 SLE patients and 22 healthy control subjects. The expression of HLA-DR and costimulatory molecules on T cells was evaluated by flow cytometry. Concentrations of serum C-reactive protein, erythrocyte sedimentation rate, anti-double-stranded DNA (anti-dsDNA) antibody, total lgG, complement 3, and complement 4 were measured. Serum cytokines and chemokines were measured by a cytometric bead array assay. Elevated frequencies of HLA-DR^+^ T cells and ICOS^+^ T cells were observed in SLE patients with positive anti-dsDNA antibodies compared with those in healthy controls (*P* < 0.001). The expression of HLA-DR^+^ T cells was positively correlated with SLEDAI (*r* = 0.15, *P* < 0.01). Furthermore, levels of serum IL-6, MCP-1, TNFRI, IL-10, IL-12, and CCL20 were higher in SLE patients compared with healthy controls. In addition, patients with hematologic manifestations displayed elevated frequencies of HLA-DR^+^ T cells and ICOS^+^ T cells. Patients with renal manifestations had a decreased frequency of TIGIT^+^ T cells. These results suggested a dysregulated T cell activity and cytokine expression profiles in SLE subjects. We also developed a chemokine and cytokine profiling strategy to predict the activity of SLE, which has clinical implication for better monitoring the flares and remission during the course of SLE and for assessing therapeutic interventions.

## 1. Introduction

Systemic lupus erythematosus (SLE) is a chronic autoimmune disease characterized by widespread immune complex formation in various organs resulting in multisystem disorders [1]. Organs such as the skin, joints, blood cells, kidneys, heart, and lungs and the nervous system are always involved. SLE affects females more frequently than males, at a ratio of about 9 : 1 [2]. Although the exact factors leading to the onset and progression of SLE have not yet been discovered, hormonal, environmental, and genetic factors are believed to be involved in the etiology of this disease [3]. While SLE is a cyclical disease, it is hard to predict its flares and remission. Thus, it is necessary to develop an accurate biomarker to evaluate the disease activity.

Given multiple immune malfunctions that evoke the diverse clinical manifestations of SLE, there is no single test available for diagnosing this disease. Overproduction of autoantibodies and disrupted regulation of multiple cytokines and chemokines are the main pathological hallmarks of SLE, which arises from T cell and antigen-presenting cell (APC) abnormalities [4]. T cell function is regulated by surface molecules such as HLA-DR, the inducible costimulatory molecule (ICOS), T cell immunoreceptor with Ig and immunoreceptor tyrosine-based inhibitory domains (TIGIT; also known as VSIG9), programmed cell death 1 (PD-1), T cell immunoglobulin, and mucin domain-containing protein 3 (TIM-3). HLA-DR, expressed on T cells, is an indicator of immunological activation [5]. Notably, accumulating evidence suggests that dynamic expression of many costimulatory and coinhibitory molecules on the surface of T cells is induced following activation [6]. ICOS is a costimulatory receptor, which induces the expression of interleukin- (IL-) 4, IL-10, and IL-21 through the PI3K signaling pathway. While in contrast, PD-1, TIGIT, and TIM-3 are coinhibitory receptors downregulating both CD4^+^ and CD8^+^ T cell responses during the T cell activation [6].

Dysregulation of chemokines and cytokines may contribute to dysfunction of immune surveillance mechanisms assumed to be able to avoid autoimmunity. T cells can be divided into T helper cell (Th) 1 (IFN-*γ*), Th2 (IL-4, IL-6, and IL-10), Th9 (IL-9), Th17 (IL-17), and regulatory T cell (Treg) (IL-10 and TGF-*β*) lineages. Studies suggested that SLE has an impact on both the Th1/Th2 and the Th17/Treg paradigm [1, 7]. Macrophage chemotactic protein- (MCP-) 1, also known as CCL2, potentially promotes migration of cells including T cells, basophils, dendritic cells, and monocytes to the site of tissue injury [8]. Previous studies demonstrated that serum levels of MCP-1 were correlated with active lupus nephritis [9]. High levels of sTNFR are indicative of frequent cleavage of the TNF receptors in some diseases, such as SLE-related autoimmune haemolytic anaemia (AIHA) [10]. CCL17 (thymus and activation-regulated chemokine, TARC) is a ligand for CC chemokine receptor 4 (CCR4), a chemokine receptor predominantly expressed by Th2 cells. It has been previously suggested that the elevation of plasma CCL17 levels is closely related to the development of SLE [11, 12]. CCL20 (MIP-3a), produced by Th17 cells, recruits inflammatory cells including lymphocytes and macrophages. Th17 response is increased via producing this chemokine. Patients with new-onset SLE have high levels of CCL20 which is correlated with disease activity [13]. However, how these molecules behave in a regulatory network in the pathogenesis of SLE awaits further clarification.

Thus, the aim of this study was to investigate the contribution of HLA-DR and costimulatory molecules on T cells together with relevant chemokines and cytokines involved to the development of SLE, so as to determine whether the expression of these moleculars are associated with disease activity and organ involvement.

## 2. Materials and Methods

### 2.1. Ethics Approval and Consent to Participate

This study was approved by the Ethics Committee of the Affiliated Hospital of Guizhou Medical University and was carried out in compliance with the Helsinki Declaration. Informed consent had been obtained from the patients' legal guardians.

### 2.2. Patients and Healthy Control Subjects

A total of 49 patients with SLE (44 females and 5 males; mean age: 34.5 ± 14.3 years) and 22 healthy control subjects (HC, mean age: 32.7 ± 11.2 years) without inflammatory or autoimmune diseases were enrolled into the study. Another 23 patients with rheumatoid arthritis (RA, mean age: 50 + 14 years) were also recruited in this study as the disease controls. The diagnosis of SLE and RA was based on the 2015 Systemic Lupus International Collaborating Clinics (SLICC) and American College of Rheumatology (ACR) revised criteria [14] and the American College of Rheumatology/European League Against Rheumatism (ACR/EULAR) 2010 rheumatoid arthritis classification criteria [15], respectively. Disease activity was assessed by the SLE disease activity index (SLEDAI). All patients were recruited from the in-patient clinic at the Division of Immunology and Rheumatology, the Affiliated Hospital of Guizhou Medical University. For the organ involvement assessment, those patients with anemia and/or thrombocytopenia were recognized as with hematologic involvement, while patients with proteinuria were identified as with renal manifestation. Blood from each individual was collected before the medicine treatments were applied. Samples were harvested in parallel into 5 ml tubes with a clot activator for harvesting serum and 5 ml tubes containing K_2_-EDTA for plasma separation. Plasma samples were harvested and stored at -80°C until respective mediator analysis. PBMCs from SLE patients, RA patients, and healthy controls were collected for analyzing the molecular phenotypes of lymphocytes.

### 2.3. Cell Surface Staining and Flow Cytometric Analysis

PBMCs from patients with SLE or RA and HC were collected and analyzed immediately for the molecular phenotypes of lymphocytes. The antibodies used for the surface marker analysis include anti-human CD3-ECD (Beckman Coulter, USA), CD4-Percp-Cy5.5, CD8-PE-Cy7, PD1-FITC, TIGIT-APC, ICOS-PE, TIM3-BV421, CD19-V500, CD3-FITC, CD8-V500, CD25-APC, HLA-DR- PE-Cy7, CD69-V450, and CD127-PE (BD Biosciences, USA). Briefly, 50 *μ*l of cells was incubated with appropriate antibodies on ice in the dark for 30 min, followed by washing in PBS. All the samples were analyzed with a Navios flow cytometer (Beckman Coulter) and Kaluza analysis software (Beckman Coulter).

### 2.4. Assessment of Autoantibodies

Serum samples were collected for autoantibody detection using the commercially available diagnostic kit (EUROIMMUN, Germany). Anti-double-stranded DNA (anti-dsDNA) antibodies were determined by indirect immunofluorescence following the instruction of the manufacturer.

### 2.5. Serum Total IgG, C-Reactive Protein, Complement 3, and Complement 4 Measurement

Total IgG, C-reactive protein (CRP), complement 3 (C3), and complement 4 (C4) in the serum from SLE patients were measured using the scattering immunotubinity measurement with an Immage 800 instrument (Beckman Coulter).

### 2.6. Erythrocyte Sedimentation Rate (ESR)

Peripheral blood from SLE patients was collected with EDTA as an anticoagulant and analyzed immediately for erythrocyte sedimentation rate (ESR) using the Westergren method examined by Roller 20 (ALIFAX, Italy).

### 2.7. Measurement of Cytokines

Cytokines in the plasma were measured by a protein antibody array methodology (RayBio^®^ Cytokine Antibody Arrays, RayBiotech, USA), which is capable of detecting 120 different cytokines from plasma with high sensitivity and specificity, according to the manufacturer's instructions. Quantification of the levels of cytokines which are correlated to the density of individual spots was carried out using Axon GenePix for image capture.

### 2.8. Cytometric Bead Array

Quantification of chemokines and cytokines in the plasma was performed with a cytometric bead array (CBA) assay (BD Biosciences). CBA was conducted with specific antibodies for IL-1*β*, TNFR1, IL-4, IL-6, IL-9, IL-10, IL-17A, MCP-1, IFN-*γ*, G-CSF, and IL-12 in accordance with the manufacturer's instructions. The fluorescence intensity was assessed on a BD ARIA III flow cytometer, followed by data analysis using FCAP Array version 3.0 software (Soft Flow, USA).

### 2.9. ELISA

CCL17 and CCL20 in the plasma were analyzed by an enzyme-linked immunosorbent assay (ELISA, R&D systems, USA) according to the manufacturer's instructions.

### 2.10. Statistical Analysis

Data were analyzed with GraphPad Prism version 5.0 (GraphPad Software, USA) and were presented as mean ± standard deviation. The significant differences between groups were determined by the unpaired *t*-test or two-way ANOVA. The Pearson method was used for correlation analysis between two variables. Logistic regression analysis (SPSS21.0 IBM) was used for multivariable association evaluation. A value of *P* < 0.05 was considered statistically significant.

## 3. Results

### 3.1. Characteristics of Study Subjects

Forty-nine patients with SLE and twenty-two HC were recruited in this study. The demographics and clinical manifestations of these patients are shown in Table 1. The majority of SLE patients (65%) were positive for anti-dsDNA antibodies. Among the patients with SLE, 84% had renal involvement, 65% had skin manifestations, and 71% had hematological involvement.

### 3.2. Elevated Expression of HLA-DR and ICOS on T Cells in Patients with SLE

**Figure 1:**
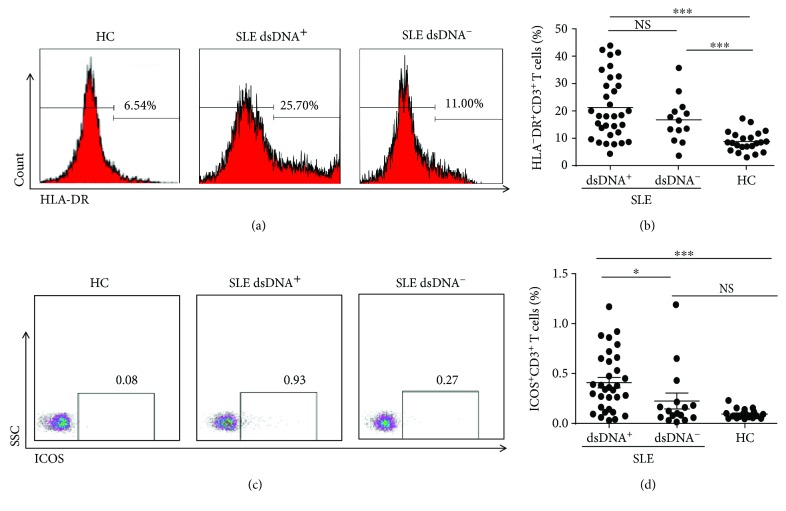
Frequencies of HLA-DR^+^CD3^+^ and ICOS^+^CD3^+^T cells in peripheral blood. Peripheral blood mononuclear cells (PBMCs) from double-stranded DNA (dsDNA)^+^ systemic lupus erythematosus (SLE) patients (*n* = 23), dsDNA^−^SLE patients (*n* = 17), and healthy control subjects (*n* = 22) were harvested and stained with appropriate flow antibodies, and the expression of HLA-DR^+^CD3^+^ and ICOS^+^CD3^+^ cells was analyzed by flow cytometry. (a, b) Representative dot plots of HLA-DR^+^CD3^+^ T cells (a) and the summarized graph (b) are displayed. (c, d) Representative dot plots (c) and pooled data (d) of ICOS^+^CD3^+^ T cells are displayed. Each dot in (c, d) represents one subject. ^∗^ *P* < 0.05, ^∗∗^ *P* < 0.01, ^∗∗∗^ *P* < 0.001, NS: no statistical significance.

T cells are documented to be critical in controlling autoimmune diseases. Therefore, we first focused on T cells and evaluated the key activation molecules, HLA-DR, and costimulatory molecules such as ICOS on CD3^+^ T cells. The frequencies of HLA-DR^+^ T cells were notably elevated in SLE patients irrespective of the presence or absence of anti-dsDNA antibodies (Figures 1(a) and 1(b), *P* < 0.001). In contrast, the ICOS expression in SLE was correlated to the anti-DNA antibodies. Those SLE subjects who produced anti-dsDNA antibodies had a higher frequency of ICOS^+^ T cells compared with those negative for anti-dsDNA antibodies and the HC (Figures 1(c) and 1(d), *P* < 0.001). When we tried to look closer into the frequencies of HLA-DR and ICOS on CD4^+^ or CD8^+^ T cells, no obvious differences were observed among these subjects (data not shown).

### 3.3. Elevated Expression of Checkpoint Molecules on CD4^+^ T Cells and CD8^+^ T Cells in Patients with SLE

**Figure 2:**
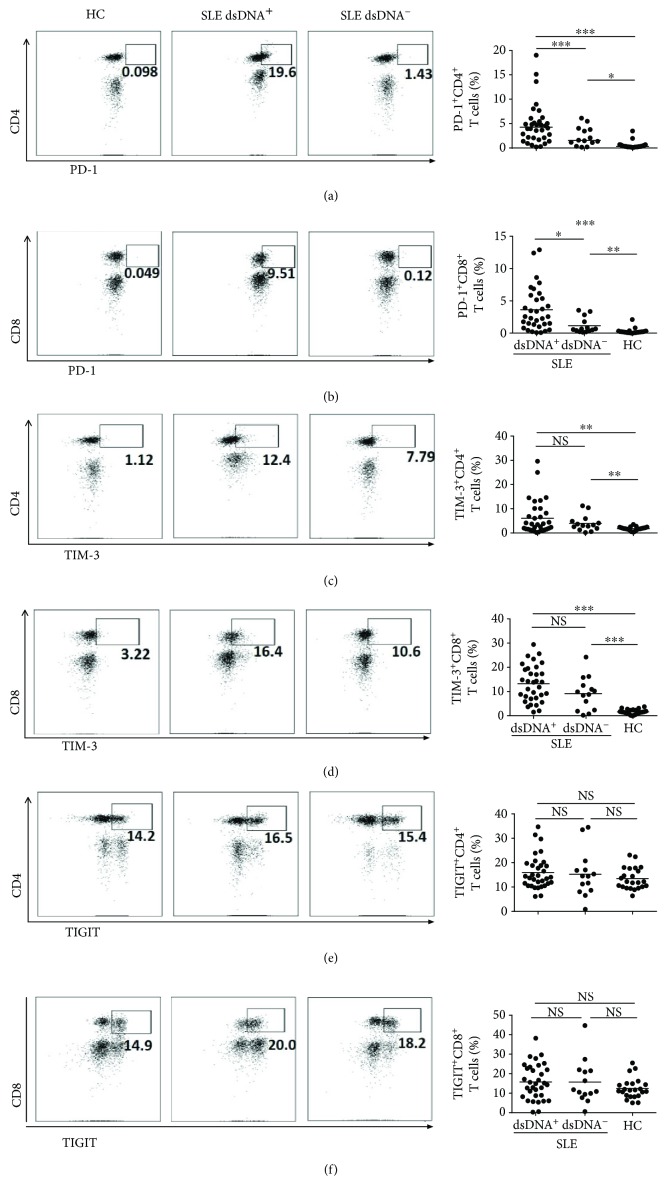
Expression levels of PD1, TIM3, and TIGIT on blood T leukocytes. PBMCs from dsDNA^+^ systemic lupus erythematosus (SLE) patients (*n* = 23), dsDNA^−^SLE patients (*n* = 17), and control subjects (*n* = 22) were harvested and stained with appropriate flow antibodies, and the expressions of PD-1, TIM-3, and TIGIT on CD3^+^ T cells were detected by flow cytometry. The flow figures and pooled data for PD-1^+^ CD4^+^ T cells (a), PD-1^+^ CD8^+^ T cells (b), TIM-3^+^CD4^+^ T cells (c), TIM-3^+^ CD8^+^ T cells (d), TIGIT^+^ CD4^+^ T cells (e), and TIGIT^+^ CD8^+^ T cells (f) are shown. Each dot in the statistical graphs represents an individual subject. ^∗^ *P* < 0.05, ^∗∗^ *P* < 0.01, ^∗∗∗^ *P* < 0.001, NS: no statistical significance.

T cells are known to upregulate the express of checkpoint molecules including PD-1, TIM-3, and TIGIT in response to variable cases, such as inflammation and infection, [16, 17] to maintain immune homeostasis. Hence, we tried to characterize the expression of these molecules on CD3^+^ T cells in the peripheral blood. SLE patients had increased frequencies of PD-1^hi^CD3^+^CD4^+^ T cells (Figure 2(a)) and PD-1^hi^CD3^+^CD8^+^ T cells (Figure 2(b)), which was even more apparent on T cells from subjects with positive anti-dsDNA antibodies (Figures 2(a) and 2(b)). A similar scenario was found for the expression of TIM-3, which was also higher on CD3^+^CD4^+^ (Figure 2(c)) and CD3^+^CD8^+^ T cell (Figure 2(d)) subsets in SLE patients, except that no difference was observed regarding anti-dsDNA antibodies positive or not. TIGIT expression was similar among SLE patients and HC (Figures 2(e) and 2(f)).

We also analyzed the data based on MFI measurements. For the majority of the molecules analyzed, these two measurement methods resulted in consistent results (Supplementary Figure 1a-1c). However, expression of TIM-3 was found to be elevated in SLE patients using the percentage analysis, whereas this enhancement disappeared when analyzed by the MFI (Supplementary Figure 1b and 1c).

The relevant molecules on peripheral T cells from patients with RA were also investigated. Essentially, both PD-1-expressing CD4^+^ T cells (Supplementary Figure 2a) and CD8^+^ T cells (Supplementary Figure 2b) were elevated in the RA subjects compared with the healthy controls, which is similar to the comparisons between SLE patients and control. However, we failed to observe increased expression of HLA-DR and ICOS on RA patient T cells (Supplementary Figure 2c).

### 3.4. Correlation of the Expression of T Cell Surface Markers with SLE Disease Activity

**Figure 3:**
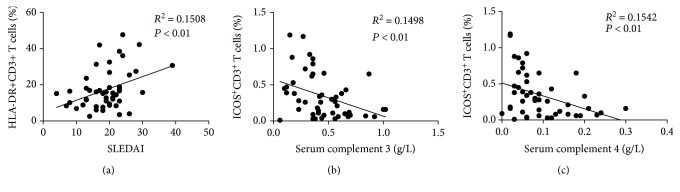
Association between the expression of HLA-DR or ICOS on T cells and the disease activity in SLE subjects. Serum complement 3 (C3) and complement 4 (C4) were measured, and the systemic lupus erythematosus (SLE) disease activity index (SLEDAI) was evaluated for each recruited patient. Correlations between the frequency of HLA-DR^+^CD3^+^ T cells and SLEDAI (a), the frequency of ICOS^+^CD3^+^ T cells, and serum complement 3 (C3) (b) or complement 4 (C4) (c) were analyzed. Each dot represents an individual patient. Correlations were evaluated with Spearman's nonparametric test. *P* < 0.05 indicates a significant difference.

We next investigated whether the expression of HLA-DR and costimulatory molecules was correlated with the SLE disease activity. HLA-DR was found to be positively correlated with SLEDAI (Figure 3(a), *r* = 0.15, *P* < 0.01). On the contrary, a negative correlation was found between ICOS and the complement components, such as C3 (Figure 3(b), *r* = −0.15, *P* < 0.01) and C4 (Figure 3(c), *r* = −0.15, *P* < 0.01). No clear correlations were observed when we compared the other surface makers studied on T cells with SLEDAI (data not shown).

### 3.5. Variation of Serum Chemokine and Cytokine Levels in SLE

**Figure 4:**
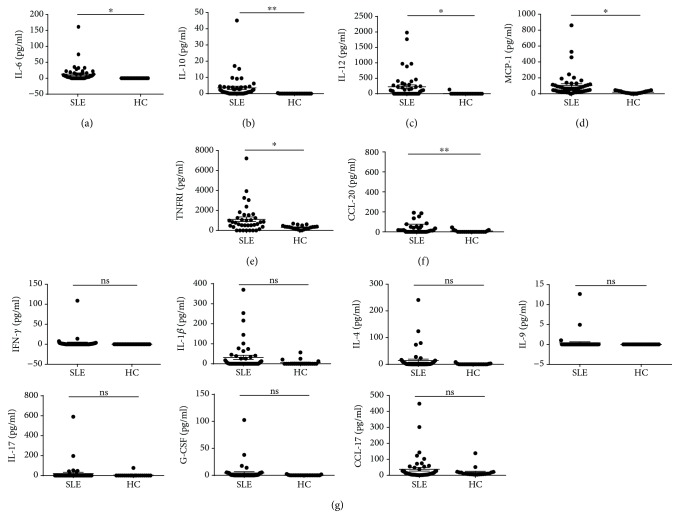
Analysis of serum cytokines and chemokines in SLE and HC. Serum from systemic lupus erythematosus (SLE) patients (*n* = 49) and control subjects (*n* = 22) was harvested for the cytokine measurement. The concentrations of serum IL-1*β*, TNFR1, IL-4, IL-6, IL-9, IL-10, IL-17, MCP-1, IFN-*γ*, G-CSF, and IL-12 were assessed by a cytometric bead array (CBA) assay. CCL-17 and CCL-20 were measured with ELISA. (a-f) Concentrations of IL-6 (a), IL-10 (b), IL-12 (c), MCP-1 (d), TNFRI (e), and CCL20 (f) in SLE patients and healthy controls were measured. (g) Concentrations of IFN-*γ*, IL-1b, IL-4, IL-9, IL-17, G-CSF, and CCL-17. Each dot represents an individual subject. ^∗^ *P* < 0.05, ^∗∗^ *P* < 0.01, ns: no statistical significance.

Next, we tried to evaluate the express pattern of circulating cytokines and chemokines. Increased levels of IL-9, MIP-3a, TARC, IL-1*β*, IL-12, MCP-1, TNFRI, G-CSF, and IFN-*γ* were observed in SLE patients based on a cytokine antibody array assay (Supplemental Figure 3). In order to further confirm this finding, cytokines and chemokines including IL-1*β*, TNFRI, IL-4, IL-6, IL-9, IL-10, IL-17A, MCP-1, IFN-*γ*, G-CSF, IL-12, CCL17, and CCL20 were quantified by CBA or ELISA. SLE patients exhibited notable increased levels of IL-6, IL-10, IL-12, MCP-1, TNFRI, and CCL20 (Figures 4(a)–4(f)). However, there were no obvious changes regarding IFN-*γ*, IL-1*β*, IL-4, IL-9, IL-17, G-CSF, or CCL-17 (Figure 4(g)), except a moderate trend of an increase in IL-1*β* or CCL17 in the SLE subjects (Figure 4(g)). Taken together, at least we can conclude that IL-6, IL-10, IL-12, MCP-1, TNFRI, and CCL20 were upregulated in the SLE patients.

The relevant cytokines in the serum of the RA patients were also measured. RA patients exhibited increased levels of several inflammation-related cytokines, including INF-*γ*, TNFR, and IL-10 (Supplementary Figure 3a, 3b and 3c). MCP-1 and CCL17, two monocyte-related chemokines, were also upregulated in subjects with RA (Supplementary Figure 3d and 3e). We failed to observe differences in the expression of other cytokines, including IL-1, IL-4, IL-6, and CCL20 as those shown in the SLE patients and HC (data not shown).

To better understand the potential associations among these cytokines and the disease, multivariable associations between the cytokines and the chemokines of SLE and those of HC were assessed through the use of logistic regression analysis. We observed that increasing concentrations of serum IL-6 and TNFRI are associated with a higher risk of SLE (Table 2).

### 3.6. Clustering Analysis of the Cytokine Data

**Figure 5:**
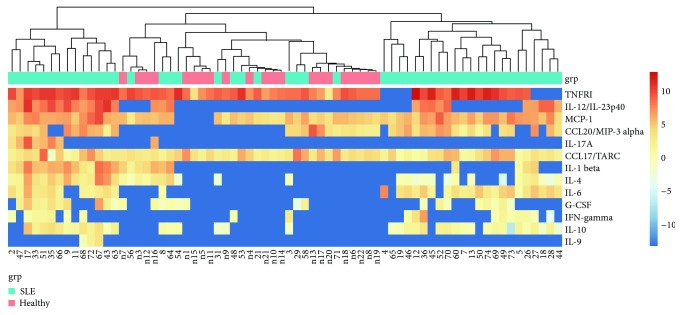
Clustering analysis on detected cytokines in SLE and HC. The concentrations of serum IL-1*β*, TNFR1, IL-4, IL-6, IL-9, IL-10, IL-17A, MCP-1, IFN-*γ*, G-CSF, IL-12, CCL17, and CCL20 in SLE patients (*n* = 49) and control subjects (*n* = 22) were assessed as explained in Figure 4, followed by hierarchical clustering analysis. The heatmap provides a qualitative evaluation of subjects in each group with regard to their cytokine and chemokine expression profiles. The heatmap legend indicates the fold change value range.

We evaluated cytokine expression patterns for the recruited SLE subjects. Cytokine profiling was carried out for each individual patient based on the 12 detected cytokines and chemokines. Cytokine signatures were found to be clustered using the hierarchical method, and three patient subgroups, namely, weak, moderate, and severe, could be identified (Figure 5). The weak group including 12 subjects exhibited a profile similar to HC, and the moderate group with 23 subjects presented a profile more deviating from HC. Fourteen subjects with a profile apparently different from HC fell into the severe group, who also exhibited a higher frequency (79%) of anti-dsDNA antibodies, a common diagnostic biomarker for SLE, in contrast to a slightly lower antibody frequency (70%) in the moderate group. Consistently, although a third of the subjects in the weak group also were positive for anti-dsDNA antibodies, half of the positive subjects expressed merely marginal levels of anti-dsDNA antibodies.

### 3.7. Correlation of Molecule Expressions on T Cells with Organ Involvement

**Figure 6:**
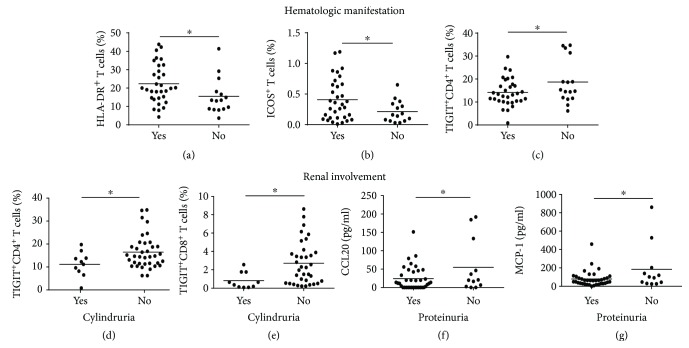
Variable expression of HLA-DR, ICOS, and TIGIT on T cells in SLE patients with organ involvement. SLE patients were divided into different groups according to their hematologic or renal manifestations. (a–c) Frequencies of HLA-DR^+^CD3^+^ T cell (a), ICOS^+^ T cell (b), and TIGIT^+^CD4^+^ T cell (c) in SLE patients with or without active hematologic manifestations. (d–g) Frequencies of TIGIT^+^CD4^+^ T cell (d) and TIGIT^+^CD8^+^ T cell (e) and level of serum CCL-20 (f) and MCP-1 (g) in patients with or without active renal manifestations. Each dot represents an individual subject. ^∗^ *P* < 0.05.

The development and progression of diseases such as SLE were always accompanied with organ dysfunction. Given the altered cell surface molecule expression and circulating cytokine levels in the SLE patients, we moved on to assess the association of these parameters with clinical manifestations. We observed a clearly increased frequency of HLA-DR^+^ T cells (Figure 6(a)) and ICOS^+^ T cells (Figure 6(b)), but comparatively decreased TIGIT^+^CD4^+^ T cells (Figure 6(c)), in those SLE patients with hematologic involvement. In addition, patients with cylindruria presented a trend of downregulated expression of TIGIT^+^ on both CD4+ T cells (Figure 6(d)) and CD8+ T cells (Figure 6(e)), and a slightly lowered circulating CCL-20 (Figure 6(f)) and MCP-1 (Figure 6(g)) was observed in those patients with proteinuria.

## 4. Discussion

In the present study, we investigated the activation-associated phenotypes of circulating T cell subsets and profiles of circulating cytokines and chemokines in SLE patients. Our data indicate a dysregulated T cell activity and a distinct pattern of cytokines partially associated with the disease development.

T cells play an important role in SLE pathogenesis [18]. HLA-DR, an indicator of immunological activation, is expressed on immune cells including T cells and is essential for T cell recognition of antigens even in the absence of APC [19]. ICOS, a member of the CD28 family, is a T cell costimulatory molecule that provides a critical signal to T cells during antigen-mediated activation [20]. Elevated expression of both HLA-DR and ICOS imply that circulating T cells in SLE patients with positive anti-dsDNA antibodies are activated. More specifically, the frequency of HLA-DR-expressing CD3^+^ T cells was associated with SLE disease severity (Figure 3(a)), suggesting a possible parameter for monitoring SLE disease progression. The expression of ICOS-expressing CD3^+^ T cells was negatively correlated with levels of complement 3 and complement 4 (Figures 3(b) and 3(c)). Enhanced expression of ICOS-expressing CD3^+^ T cells may imply that the SLE patients are at a more severe inflammatory status.

To further assess the expression of costimulatory molecules on T cell subsets, we investigated the expression of PD-1, TIM-3, and TIGIT on T cells and showed that the frequencies of PD-1 or TIM-3-expressing CD3^+^CD4^+^ T cells or CD3^+^CD8^+^ T cells were significantly increased in those SLE patients with positive anti-dsDNA antibodies compared with the HC. These results suggest that SLE is characterized by dysregulated activation of T cells, which is consistent with previous studies [21]. The expression of checkpoint molecules such as PD-1 and TIGIT have been reported to be dampened on immune cells in SLE patients [22, 23]. However, in our study, SLE subjects seemed to exhibit increased levels of PD-1, similar to what has been just revealed in systemic sclerosis [24] and another study showing that SLE patients exhibited increased frequencies of circulating PD1^+^ICOS^+^ T follicular helper cells and PD1^+^ICOS^+^ T memory cells [25], possibly suggesting a feedback mechanism of the immune system. These inhibitory receptors could have failed to suppress overwhelming T cell activation as a result of upregulation of other activation pathways.

Our study investigated the expression of these molecules on CD4^+^ T cells and CD8^+^ T cells separately. Most of previous studies focused on analyzing the expression of coinhibitory and costimulatory molecules on the surface of PBMCs. Furthermore, we recruited patients in Guizhou Province, a remote southwestern province in China, where ethnic diversity is prominent (different from Han Chinese). Based on our data, we suggest that expression of HLA-DR is a more important marker than any other cytokines and surface markers to predict the SLE risk.

Dysregulated activation of T cells might lead to an aberrant cytokine expression profile [26]. We found that circulating IL-6 and IL-10 levels were higher in patients with SLE than those in healthy controls. In contrast, IFN-*γ* levels were similar between these two groups, suggesting an imbalanced Th1/Th2 ratio in peripheral blood. This is in line with previous findings suggesting that SLE is a Th2-polarized disease because of the production of autoantibodies specific for self-antigens [27]. More importantly, inflammatory cytokines such as IL-12, MCP-1, TNFRI, and CCL20 from SLE patients were upregulated (Figures 4(c)–4(f)). Of interest, a group of 12 factors were screened from the cytokine antibody array data to assess the disease activity of SLE. We noticed that these 12 factors can potentially reveal SLE disease severity after clustering analysis. Thus, our work provides a novel insight into establishment of an improved profile to predict disease severity.

As a systemic autoimmune disease, organs such as the skin, joints, blood cells, kidneys, heart, and lungs and the nervous system are always involved in SLE patients. Cytokines, chemokines, and costimulatory molecules expressed on T cells have been implicated in the pathophysiology of lupus nephropathy (LN) and hematologic manifestations of SLE. Patients with hematologic manifestations displayed elevated frequencies of HLA-DR^+^ T cells and ICOS^+^ T cells, suggesting potential contribution of T cell activation to the hematologic involvement. Relatively fewer TIGIT^+^CD4^+^ T cells in those subjects with hematologic manifestations support the dampened homeostasis maintenance, which is further highlighted by the observation that patients with cylindruria also had fewer TIGIT^+^CD4^+^ T cells and TIGIT^+^CD8^+^ T cells, suggesting the important role of TIGIT in the function maintenance of relevant organs. CCL20 [28] and MCP-1 [29] can recruit monocytes and dendritic cells into local tissues involved and promote pathological progression in SLE. An interesting finding is that SLE patients collectively demonstrated increased levels of CCL20 and MCP-1 in the circulation (Figure 4), but a trend of lower levels of these two chemokines was observed in those SLE patients with renal involvement. A plausible explanation for this finding might be that these chemokines are concentrated in the renal tissues so as to maximize the local recruitment of the immune effector cells. Although most patients are multiorgan involvement rather than single organ involvement, which may affect the variations displayed, there is a caveat in these analyses because we did not exhaust the clinical examinations on these patients and we do not know if these patients (including those classified as “absence of organ involvement”) had any other unknown organ involvement.

Our study showed that patients with either SLE or RA exhibited, to some degree, similar cellular and humoral responses, including, for example, elevated frequencies of PD-1-expressing T cells, several inflammatory cytokines, and chemokines important for monocytes, which is not surprising. Both genetic and environmental factors demonstrate potential impacts on the development of autoimmune diseases, suggesting that overlapping mechanisms and clinical manifestations exist among different autoimmune diseases, such as SLE and RA, although to different extents. For example, similar Treg-related cellular responses during development of the diseases [30] and shared HLA locus associated with autoantibodies [31] have been reported in patients with either SLE or RA. Elevated anti-HLA antibodies have been detected among parous females with SLE or RA but may be particularly correlated to disease severity among SLE subjects [32].

The limitation of the current study is that we did not follow the patients for an extended period of time, which may substantiate the functional correlation.

## 5. Conclusion

In conclusion, our data suggest dysregulated activation of T cells and abnormal cytokine expression profiles in SLE. In particular, we show that HLA-DR^+^ T cell subsets are positively correlated with SLEDAI, and we have developed a chemokine and cytokine profile to predict the activity of SLE, which may have clinical implications for monitoring the flares and remission during the course of SLE. A more in-depth understanding of the regulation of these chemokines and cytokines in SLE may shed light on future therapeutic development by targeting these biomarkers.

## Figures and Tables

**Table 1:** Clinical manifestations and clinical features of SLE patients at the time of the study.

Characteristics	SLE (*n* = 49)
Age (years)	34.5 ± 14.3
Female : male	44 : 5
Clinical manifestations no. (%)	
Malar rash/discoid rash	32 (65.3)
Photosensitivity	16 (32.7)
Oral ulcer	12 (24.5)
Arthritis/arthralgia	38 (77.6)
Autoimmune hemolytic anemia	35 (71.4)
Leukopenia	18 (36.7)
Lymphopenia	34 (69.3)
Immune thrombocytopenia	23 (46.9)
Renal involvement	41 (83.7)
Neurological involvement	6 (12.2)
SLEDAI	19.2 ± 6.7
Serological features (%)	
Anti-dsDNA Abs	32
Serum C3 (g/L)	0.45 ± 0.23
Serum C4 (g/L)	0.09 ± 0.07

Abbreviations: SLE: systemic lupus erythematosus; SLEDAI: SLE disease activity index; dsDNA: double-stranded DNA; C3: complement 3; C4: complement 4.

**Table 2:** Multiple logistic regression analysis results on the potential factors associated with SLE.

Risk factor	OR	CI (95%)	*P* value
IL-6	1.673	1.013-2.764	0.044
TNFRI	1.003	1.006-2.764	0.027

OR: odds ratio; CI: confidence interval

## Data Availability

All data generated or analyzed during this study are included in this published article (and its supplementary information files).

## Supplementary Materials

Supplementary 1 Figure 1: expression level of HLA-DR, ICOS, PD-1, TIM-3, and TIGIT on peripheral T cells.

Supplementary 2 Figure 2: expression level of HLA-DR, ICOS, PD-1, TIM-3, and TIGIT on T lymphocytes in RA and HC.

Supplementary 3 Figure 3: analysis of serum cytokines and chemokines in RA and HC.

Supplementary 4 Figure 4: qualitative evaluation of samples analyzed with protein array.
